# Water-Bath Stunning Efficiency, Welfare Indicators, and Carcass Quality in Taiwanese Red-Feathered Native Chickens

**DOI:** 10.3390/vetsci13030273

**Published:** 2026-03-16

**Authors:** Pei-Tsen Lin, Penpitcha Supapaiboonkit, Yi-Tse Hsiao, Fang-Chia Chang, Yi-Chun Lin

**Affiliations:** 1 Department of Animal Science, National Chung Hsing University, Taichung 40227, Taiwan; linda202lin@gmail.com (P.-T.L.); penpitcha.sup@gmail.com (P.S.); 2 Department of Veterinary Medicine, National Taiwan University, Taipei 10617, Taiwan; ythsiao@ntu.edu.tw (Y.-T.H.); fchang@ntu.edu.tw (F.-C.C.); 3 The iEGG and Animal Biotechnology Research Center, National Chung Hsing University, Taichung 402, Taiwan

**Keywords:** animal welfare, slaughter, electrical stunning, electroencephalograms, body composition, carcass quality

## Abstract

Electrical water-bath stunning remains the predominant method used in commercial poultry slaughter worldwide yet its effectiveness and welfare implications may vary among breeds. Taiwanese red-feathered chickens differ from commercial broilers in growth rate and body composition, which may influence their response to electrical stunning. This study investigated the relationships between electrical stunning conditions, electroencephalographic (EEG) indicators of unconsciousness, behavioural reflexes, and carcass quality in Taiwanese red-feathered chickens. A total of 200 female chickens were subjected to direct-current water-bath stunning at 80, 100, 120, 140, or 160 V for 7 s. EEG activity and physical indicators of consciousness were assessed during the first 40 s after stunning, and carcass defects were evaluated post-mortem. Of the 200 birds initially evaluated, EEG data from 153 birds met predefined signal quality criteria and were included in the final analysis. EEG-defined unconsciousness was more frequent and lasted longer at higher voltages (140–160 V), although intermediate voltage levels (e.g., 120 V) did not follow a strictly linear trend. Corneal reflex and spontaneous eye blinking were strongly associated with EEG-based unconsciousness, supporting their use as practical on-site welfare indicators. At the lowest voltage (80 V), birds with higher abdominal fat percentages were more likely to be effectively stunned. In contrast, no statistically significant associations between abdominal fat percentage and stunning effectiveness were observed at 100–160 V. However, higher voltages were also associated with an increased prevalence and severity of carcass defects. These findings suggest that stunning conditions or commercial broilers may not ensure effective unconsciousness in Taiwanese red-feathered chickens. Corneal reflex and spontaneous eye blinking provide reliable, welfare-relevant indicators of unconsciousness under field conditions. Electrical settings must be carefully balanced to achieve effective stunning while minimising adverse welfare outcomes associated with excessive neuro-muscular responses.

## Simple Summary

Electrical stunning is widely used to make chickens unconscious before slaughter, but previous studies have shown that differences among breeds, including growth rate and body composition, may influence electrical resistance and stunning effectiveness. Taiwanese red-feathered native chickens grow more slowly and differ from commercial broilers in body weight, abdominal fat deposition, and musculoskeletal conformation, which may influence electrical resistance and their response to stunning. In this study, we examined the effects of different voltage levels (80–160 V) applied using a direct-current water-bath stunner on unconsciousness, behavioural responses, and carcass quality in Taiwanese red-feathered chickens. Electroencephalography (EEG) recordings were used to confirm loss of consciousness and were compared with simple physical signs such as eye blinking and eye reflexes that are commonly checked in slaughterhouses. Higher voltage settings (140–160 V) resulted in a greater proportion of birds showing EEG-defined unconsciousness and suppression of corneal reflex and spontaneous eye blinking, although intermediate voltage levels did not show a strictly linear increase in stunning effectiveness. However, these higher voltages were also associated with an increased prevalence and severity of scored carcass defects, including wing haemorrhage and red wingtips. At very low electrical settings, chickens with higher body fat were more likely to be effectively stunned. These results show that electrical stunning conditions designed for commercial broilers may not always ensure good welfare in native chickens. Simple indicators such as eye blinking and eye reflexes can help workers judge unconsciousness on the slaughter line, but electrical settings must be carefully balanced to protect both animal welfare and meat quality.

## 1. Introduction

Electrical water-bath stunning remains the predominant method used in commercial poultry slaughter worldwide [1,2,3], yet its effectiveness and welfare implications may vary among breeds [4]. For example, Taiwanese red-feathered chickens differ from commercial broilers in growth rate and body composition [5], which may influence their response to electrical stunning. In Taiwan, the main breeds of meat-type broilers are commercial broilers, which have an annual production of 180 million, and native chickens, which have an annual output of 175 million, as reported by the Ministry of Agriculture, Executive Yuan R.O.C. (Taiwan) [6]. Among the native types, Taiwanese red-feathered native chickens are a slow-growing meat strain typically reared for about 12 to 14 weeks to reach market weights of 2.3 to 3.0 kg [7,8,9]. These chickens are highly valued by consumers for their flavour and texture and are commonly used in traditional Chinese cooking methods such as boiling and simmering. In contrast, commercial broiler lines usually reach a market weight of roughly 1.8 kg in only 5 to 6 weeks [7,10], reflecting substantial genetic and physiological differences between these two production types. Differences in growth rate and slaughter age are ac-companied by differences in body composition, including proportions of muscle, fat, and bone.

In commercial slaughter plants, pre-slaughter stunning is a key step for ensuring both animal welfare and carcass quality. In Taiwan, poultry slaughter predominantly relies on electrical water-bath stunning, in which birds are shackled and their heads immersed in electrified water before neck cutting. According to European Union welfare regulations for the protection of animals at the time of killing, birds subjected to electrical stunning must be rendered insensible or unconscious and must not regain consciousness before slaughter [11]. For commercial broilers, the minimum recommended currents are 100 mA at frequencies up to 200 Hz, 150 mA at 200–400 Hz, and 200 mA at 400–1500 Hz [11]. These thresholds are intended to produce a generalised epileptiform EEG or a pro-found suppression of brain activity, indicating loss of consciousness, while minimising the risk of inadequate stunning. However, these standardised parameters are primarily optimised for commercial broilers and may not be appropriate for the unique physiological profile of Taiwanese red-feathered native chickens.

Although current animal welfare research increasingly explores alternative methods such as controlled atmosphere stunning (CAS; gas stunning) and constant-current electrical systems [12,13,14,15], conventional electrical stunning remains the predominant method used for poultry slaughter in most countries [3,16]. Consequently, evaluating the welfare outcomes of electrical stunning under field conditions remains highly relevant, particularly for poultry breeds that differ markedly from commercial broilers.

However, the electrical parameters used in water-bath systems, including voltage, current, frequency, and waveform, interact with bird-related factors such as body weight, fat content, feather condition, and skull structure [17,18]. Birds with higher electrical resistance may receive insufficient current at a given voltage, whereas birds with lower resistance may be overexposed [19].

The efficacy of water-bath stunning is governed by the biophysical relationship described by Ohm’s Law (I = V/R). While the induction of unconsciousness depends on the current intensity (I), to achieve effective stunning results [15,20,21], most industrial systems in Taiwan and globally operate under a constant voltage (CV) regime [22]. In such systems, the actual current delivered is inversely proportional to the bird’s bio-impedance (R) [23,24].

Biological impedance is highly sensitive to tissue composition. Skeletal muscle acts as a primary conductor due to its high water content (70–75%) and abundance of electrolytes. Conversely, adipose tissue is a biological insulator with a resistivity 10 to 20 times higher than muscle [25].

Native chickens differ from commercial broilers not only in growth rate and slaughter age but also in body composition and conformation [7,10]. These differences are likely to alter electrical resistance and current distribution in the water bath and may influence both stunning effectiveness and the prevalence of carcass defects. Therefore, stunning conditions optimised for commercial broilers may not be appropriate for Taiwanese red-feathered native chickens.

The effectiveness of electrical stunning can be assessed using both physiological and behavioural measures. Electroencephalography (EEG) provides a direct indicator of brain function and is considered the most objective method for distinguishing conscious from unconscious states. In broilers, unconsciousness has been defined as an isoelectric EEG or a profound reduction in brain power to less than 10% of the pre-stun level [13,26]. Under commercial conditions, however, EEG is rarely available, and slaughterhouse staff usually rely on physical reflexes such as corneal reflex, spontaneous eye blinking, wing flapping, tonic–clonic activity, neck tension, and rhythmic breathing to judge whether stunning has been effective. Identifying which behavioural indicators best reflect EEG-defined unconsciousness in native chickens is therefore critical for practical welfare assessment on the slaughter line.

At the same time, electrical parameters that improve stunning efficacy may also affect carcass quality [27]. High voltages and currents can increase the incidence of carcass defects such as wing and breast haemorrhage, red wingtips, and red pygostyle, which lead to downgrading and economic losses [28]. Conversely, low electrical settings may reduce these defects [29] but risk ineffective stunning and compromised animal welfare [26]. Balancing stunning effectiveness with carcass quality is particularly important in the Taiwanese market, where whole native chickens are often sold and visible defects directly influence consumer acceptance.

Therefore, this study aimed to evaluate the welfare implications of water-bath stunning in Taiwanese red-feathered native chickens by linking EEG-defined unconsciousness with behavioural indicators and carcass outcomes. The findings are intended to support veterinary assessment of unconsciousness and stunning effectiveness under field conditions and to inform animal welfare-relevant outcomes.

## 2. Materials and Methods

### 2.1. Animals and Experimental Design

A total of 200 female red-feathered native chickens aged 10 weeks were obtained from a commercial farm and randomly allocated to five homogeneous treatment groups (40 birds per group), each subjected to a different voltage treatment. All stunning and slaughter procedures were carried out at the university slaughter facility of National Chung Hsing University.

Birds were stunned individually in a water bath for 7 s using a direct current set at 80, 100, 120, 140 or 160 V. The results of the current depended on the electrical resistance of each chicken and were recorded for every stun. To characterise the electrical output of the system, the waveform was measured using a digital oscilloscope (DSOX1202A, Keysight Technologies, Santa Rosa, CA, USA). The measurements indicated that the output consisted of a unidirectional direct current (DC). At nominal voltage settings of 80–140 V, the measured root mean square (RMS) voltages were approximately 82.9 V, 100.9 V, 120.5 V, and 140.0 V, respectively. The signals showed minor voltage ripple, with peak-to-peak amplitudes ranging from approximately 10.9 V to 24.1 V. The waveform at the nominal 160 V setting was not recorded during the oscilloscope measurements. Representative oscilloscope waveforms at different voltage settings are provided in Supplementary Figure S1.

Electroencephalography (EEG) and physical reflexes were recorded at three time points after stunning: P1, 10 to 20 s; P2, 20 to 30 s; and P3, 30 to 40 s. Electrode implantation for EEG evaluation was performed at 11 weeks of age, and the chickens were slaughtered at 12–13 weeks of age. Due to unavoidable logistical constraints, birds assigned to the 120 V treatment were slaughtered approximately 5 days later than birds in the other voltage groups. All birds remained within the typical commercial slaughter age range for Taiwanese red-feathered native chickens. The average body weight before slaughter was 2.76 ± 0.37 kg. Feed was withdrawn 9–10 h prior to slaughter. The experimental protocol was reviewed and approved by the Institutional Animal Care and Use Committee of National Chung Hsing University (IACUC 111-008).

### 2.2. Effectiveness Evaluation

Stunning effectiveness was assessed using EEG recordings and physical reflexes obtained via electrodes implanted into the skull of each chicken. After surgery, the chickens were allowed a 1-week recovery period. Birds were visually examined by trained personnel to assess general health status, including posture, respiration, alertness, and absence of bleeding or abnormal behaviour. Only birds that appeared clinically normal were included in the subsequent stunning trials. One week after electrode implantation, electrode placement and function were verified. For this test, birds were restrained by the wings and anesthetised with 5% isoflurane in oxygen at a flow rate of 0.8 L/min, and their behavioural responses were observed to confirm loss of consciousness. Once birds were unconscious, the isoflurane concentration was reduced to 4%, and EEG was recorded for 2 min using an IX-BIO4 biopotential amplifier (iWorx Systems Inc. IX-BIO4 biopotential amplifier. Dover, NH, USA). EEG signals were also recorded while birds were awake, and recordings obtained during conscious and anaesthetised states were compared qualitatively to confirm that the implanted electrodes accurately reflected brain activity in both conditions. Physiological and behavioural responses were monitored throughout to ensure that birds remained in a stable, unconscious state during anaesthesia.

Before slaughter, baseline EEG was recorded for 2 min. Post-slaughter EEG was recorded for 2 min within 5 s after water-bath stunning. Signals were recorded and analysed using LabScribe v4. software (iWorx Systems Inc. LabScribe v4: data recording and analysis software. Dover, NH, USA) and processed by fast Fourier transform (FFT). Six segments of pre-stun EEG were divided into three segments corresponding to P1–P3. Birds were classified as unconscious when post-stun brain power was <10% of the corresponding pre-stun value [13,26]. EEG recordings were obtained from 200 birds. However, recordings with unstable baseline signals, excessive artefacts, electrode detachment, or incomplete data acquisition were excluded according to predefined quality criteria. After quality control, 153 recordings were deemed suitable for statistical analysis.

During EEG recording, physical reflexes were assessed within the first 40 s after stun. A positive response suggested a reaction to the stimulus, whereas a negative response suggested no reaction. After stunning, birds were assessed for spontaneous eye blinking without stimulation and corneal reflex, elicited by gently touching the cornea with a feather or finger to provoke eyelids closure or movement of the nictitating membrane. Wing flapping was defined as continuous, rapid flapping of the wings.

Neck tension was evaluated by palpating the neck muscles and observing muscle stiffness and head raising. Tonic–clonic seizure was identified as rigid backward bending of the neck and tucked wings, sometimes accompanied by small and rapid muscular con-tractions, followed by relaxation of the body. Response to a pain stimulus was assessed by pinching the comb and observing head shaking or struggling. Rhythmic breathing was assessed by observing the regular movement of the chest and cloaca. The mean duration of unconsciousness was calculated only for chickens that were classified as stunned successfully. All behavioural observations (reflex testing and seizure assessment) were performed by a single trained observer (one of the authors) following a standardised protocol.

EEG and behavioural responses were monitored for 40 s after stunning to capture both the immediate induction of unconsciousness and potential recovery prior to exsanguination, consistent with previous EEG-based poultry stunning protocols [13,21,26,30]. The 7 s stun duration reflected the preset exposure time of the commercial DC water-bath system and falls within the range commonly used in commercial practice. EEG recording commenced at 10 s post-stun to allow safe attachment and stabilisation of the recording equipment. This ensured acquisition of stable and interpretable EEG signals. Consequently, the 0–10 s interval was not included in the analysis.

### 2.3. Carcass Evaluation and Fat Content

**Figure 1:**
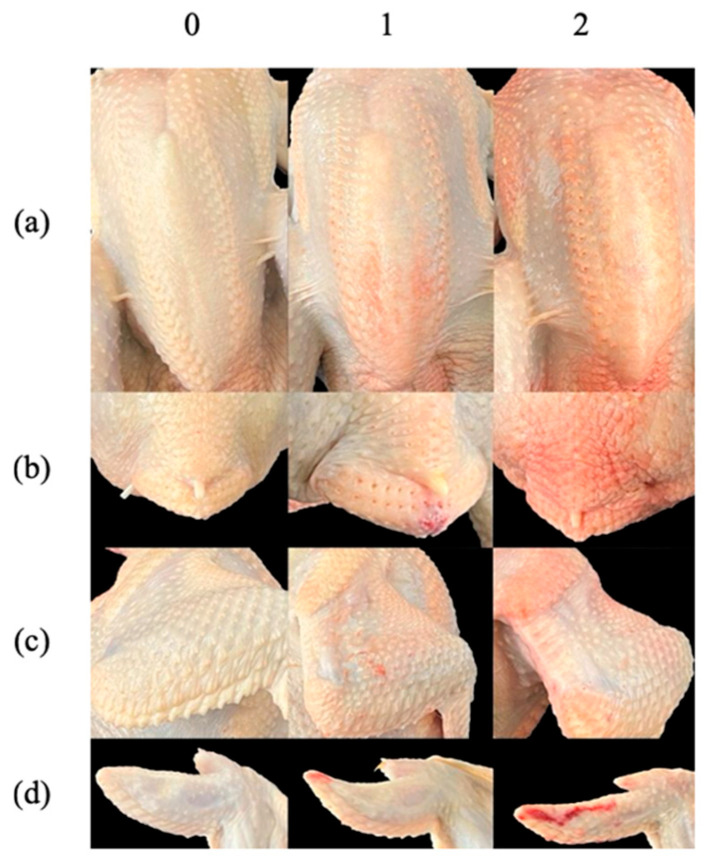
Evaluation of carcass quality through appearance defects (**a**) BHM: Breast haemorrhage, (**b**) RPG: Red pygostyle, (**c**) WHM: Wing haemorrhage, and (**d**) RWT: Red wingtips. Score of 0 indicates absence of damage; 1 indicates slight damage when defects show less than or equal to 50% in the observed area; and 2 indicates severe damage when defects show greater than or equal to 50% in the observed area.

Following exsanguination and automated plucking at the university slaughter facility, carcasses were photographed and video footage was recorded from four perspectives to document external defects. Each carcass was visually scored for external quality using a 0–2 scale based on the presence and extent of breast haemorrhage, wing haemorrhage, red wingtips, and red pygostyle, as illustrated in Figure 1. A score of 0 indicated absence of damage; 1 indicated slight damage, when defects covered ≤50% of the observed area; and 2 indicated severe damage, when defects covered ≥50% of the observed area. This more sensitive scoring system was applied because, based on their general appearance, all carcasses would have met United States Department of Agriculture Grade A criteria.

Carcass quality was graded independently by three trained assessors who were blinded to the voltage treatment applied to each bird. For each carcass and each defect category, the final score was calculated as the mean of the three assessors’ scores.

Subsequently, the carcasses were manually eviscerated, and all viscera were removed. Abdominal fat located around the proventriculus, and along the gizzard to the cloaca [31], was carefully dissected and subsequently weighed.

### 2.4. Statistical Analysis

All statistical analyses were performed using SPSS Statistics 29.0.1.0 (IBM Corp., Armonk, NY, USA). Chi-square tests, Pearson correlation analysis, binary logistic regression, one-way analysis of variance (ANOVA) with post hoc tests, and Spearman’s rank correlation were used as appropriate. Voltage (80–160 V) and the corresponding current were included as explanatory variables where applicable. The individual bird was considered the experimental unit. Differences were considered statistically significant at *p* ≤ 0.05 and as a trend at 0.05 < *p* < 0.10.

## 3. Results

The mean and standard deviation of the electrical conditions and body composition are summarised in Table 1. Birds stunned at 120 V had the greatest body and carcass weights (*p* < 0.05), followed by those stunned at 140 V, whereas birds in the 80 and 100 V groups had the lowest values. The 160 V group showed intermediate body and carcass weights that were generally similar to those of the lower-voltage groups. Abdominal fat weight was higher at 120 and 140 V than at the other voltages, with no significant difference between these two groups (*p* < 0.05).

Mean current differed among all voltage treatments and increased with voltage except for the 120 V group, with the highest current observed in the 160 V group and the lowest in the 80 V group (*p* < 0.05). Notably, the 120 V group exhibited a lower mean current than the 100 V group, indicating higher apparent electrical resistance (R = V/I) at this voltage level. Stunning time was slightly shorter at 100 and 120 V than at 80, 140, and 160 V. The duration of unconsciousness tended to increase with voltage. Birds stunned at 160 V remained unconscious longest, those at 100 and 120 V had the shortest unconscious times, and birds at 80 and 140 V showed intermediate values (Table 1).

Pearson’s correlation coefficients describing relationships among body composition variables and unconscious time are shown in Table 2. Abdominal fat weight was positively correlated with body weight (r = 0.656, *p* < 0.001), and carcass weight was strongly correlated with body weight (r = 0.947, *p* < 0.001) and moderately correlated with abdominal fat weight (r = 0.575, *p* < 0.001). The fitted linear regression models were Yi = −0.121 + 0.083Xi and Zi = 0.212 + 0.739Xi, where Xi is the body weight (kg), Yi is the abdominal fat weight (kg), and Zi is the carcass weight (kg). No significant correlations were detected between body composition (body weight, abdominal fat weight, or carcass weight) and duration of unconsciousness (Table 2).

### 3.1. EEG-Defined Unconsciousness

EEG recordings were successfully obtained from 153 chickens (32, 29, 20, 36, and 36 in the 80, 100, 120, 140, and 160 V groups, respectively, shown in Table 3). At P1 (10–20 s post-stun), the proportion of unconscious chickens was highest in the 160 V group (81%), followed by the 140 V group (75%). At P2 (20–30 s post-stun), the 160 V group still showed the highest proportion of unconscious birds (78%), whereas the proportion in the 140 V group declined to 39%. At P3 (30–40 s post-stun), the proportion of unconscious birds was below 50% in all groups, indicating progressive recovery of consciousness over time. Across all time periods, unconsciousness rates in the 80, 100, and 120 V groups remained below 50%.

Epileptiform EEG, a strong indicator of immediate unconsciousness, was absent at 100 V and 120 V but prominent at 140 V and 160 V. As shown in Table 4, increasing voltage levels from 80 V to 160 V was associated with a greater number of birds classified as unconscious, particularly at P1 and P2. At P1, the number of unconscious chickens increased from 16 at 80 V to 29 at 160 V (*p* < 0.001 for voltage; *p* = 0.003 for current). Similarly, at P2, the number of unconscious birds also increased significantly with voltage (*p* < 0.001), although the effect of the current was not statistically significant (*p* = 0.316). At P3, a voltage-dependent trend in unconsciousness was observed (*p* = 0.043), but with greater variability among treatments and no significant effect of the current (*p* = 0.073).

### 3.2. Behavioural Responses Following Stunning

**Figure 2:**
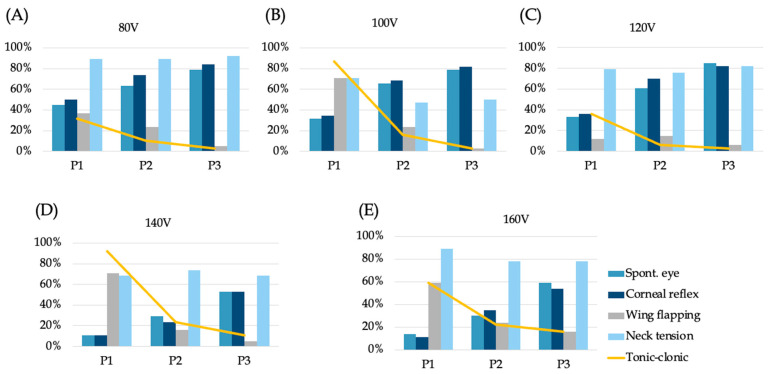
Positive behaviour percentages in each group. (**A**) 80 V, (**B**) 100 V, (**C**) 120 V, (**D**) 140 V, and (**E**) 160 V. Periods: P1, 10 to 20 s; P2, 20 to 30 s; P3, 30 to 40 s.

Every bird showed rhythmic breathing, and none responded to comb pinching. Therefore, these two indicators are not presented in Figure 2. Spontaneous blinking and corneal reflex followed similar patterns across voltage groups. At P1, positive responses for spontaneous eye blinking were least frequent in the 140 V group (11%), followed by the 160 V group (14%). The percentage of positive responses for corneal reflex responses in both groups was also 11%. At P2, the positive response rate for the 80, 100, and 120 V groups exceeded 50%, indicating that within 30 s after the stun, 50% of chickens regained consciousness. At P3, more than 50% of chickens in all groups showed positive responses for eye reflexes, particularly in the 120 V group; the positive rates for both spontaneous blinking and corneal reflex exceeded 80%, suggesting that most birds were conscious by this time.

The results of the wing flapping responses are shown in Figure 2. The positive responses in all groups except 120 V exceeded 50% during P1, with no significant differences found between these groups. The percentage of positive responses for neck tension responses was more than 50% in all treatment groups at every time point. Tonic–clonic activity at P1 was particularly frequent at 100 and 140 V (87% and 92%, respectively). Overall, these behavioural results suggest that eye reflexes were more effectively suppressed at higher voltages, whereas wing flapping, neck tension, and tonic–clonic activity showed more complex patterns in relation to voltage and current.

The associations between individual behavioural responses and electrical conditions are summarised in Table 5. Voltage was significantly associated with most behavioural indicators, whereas associations with current varied among responses.

### 3.3. Body Composition and Stunning Effectiveness

To investigate the relationship between body composition and stunning effectiveness, stunned effectively was defined as birds with an isoelectric EEG in the 2–30 Hz band at all three time points (P1–P3). Spearman’s rho coefficients describing the association between stunning effectiveness and abdominal fat weight percentage (AFW%) are shown in Table 6. Stunning effectiveness was not associated with AFW% in the 100, 120, 140, or 160 V groups (*p* > 0.05). In contrast, in the 80 V group, stunning effectiveness showed a moderate positive correlation with AFW% (rho = 0.47, *p* < 0.01), suggesting that birds with relatively higher abdominal fat percentages were more likely to be effectively stunned at this low voltage.

### 3.4. Carcass Defects

Only 11 out of 186 carcasses were completely free of defects. The distribution of carcass defect scores (0–2) for each voltage group is shown in Table 7. In general, defects were often absent in carcasses from the 80, 100 and 120 V groups than in those from the 140 and 160 V groups. A stunning voltage of 100 V appeared to reduce the incidence of wing haemorrhage and red wingtips but was associated with a slight increase in low-grade red pygostyle score. The highest proportion of severe damage (score 2) for red wingtips and red pygostyle was observed at 120 V. Wing haemorrhage and red wingtips showed the highest percentage of slight damage (score 1) at 160 V, indicating that higher voltages tended to increase the incidence and severity of carcass defects.

## 4. Discussion

### 4.1. Electrical Stunning and EEG-Defined Unconsciousness

Water-bath stunning uses electricity to interfere with or block the normal brain activity of animals, inhibiting or causing loss of brain function and consciousness. The main electrical parameters that determine the induction of unconsciousness are voltage, current, frequency, and current waveform.

While alternative stunning methods such as gas stunning are actively investigated in animal welfare research, electrical stunning continues to be widely applied in commercial poultry slaughter systems in many countries. In this context, the present findings provide veterinary- and welfare-relevant evidence for assessing stunning effectiveness during poultry slaughter. These findings are particularly relevant within the preslaughter chain, where restraint, stunning, and the verification of unconsciousness are critical control points for animal welfare.

With respect to EEG, 160 V (average current of 130.5 ± 24.7 mA) produced unconsciousness in 81% of chickens during P1, of which 76% showed suppressed EEG readings and 24% showed epileptiform EEG. Birds in the 160 V group also maintained the highest percentage of unconscious EEG in P2 and P3. At 140 V (average current was 117.1 ± 25.3 mA), 75% of chickens were unconscious at P1; within this group, 4% had suppressed EEG and 96% had epileptiform EEG. However, this condition dropped to 39% and 28%, respectively, during P2 and P3, indicating that the chickens in this group showed that birds often transitioned from an epileptiform EEG pattern to recovery within 30–40 s after stunning. In contrast, average currents in the 80, 100, and 120 V groups were below 100 mA, and the overall unconsciousness rate remained below 50% across all periods, indicating that these voltage–current combinations were insufficient to reliably induce unconscious-ness. Overall, using higher voltages or achieving a minimum current of approximately 120 mA increased the proportion of unconscious birds, indicating that both voltage and current influence the depth and duration of unconsciousness. Although higher voltages increased the likelihood and duration of EEG-defined unconsciousness, they were also associated with a higher prevalence of carcass defects, which may reflect intense neuro-muscular activity and raise welfare concerns if excessive.

Previous work has highlighted that stunning effectiveness depends on the interaction between current intensity and electrical waveform characteristics, particularly frequency in alternating or pulsed systems. Prinz et al. [30] found that at the same voltage, a pulsed direct current (DC) produced lower currents and a lower proportion of unconscious chickens than an alternating current (AC). A minimum current of 130 mA was needed for a stun using pulsed DC systems to cause unconsciousness. In the AC system, Prinz et al. [32] recommended a minimum of 120 mA at 400 Hz to achieve a high proportion of unconscious birds. If a low frequency is combined with a current above 100 mA, chickens may fail to regain consciousness and die, indicating an irreversible stun. Raj et al. [33] proposed that minimum currents of 100 mA with 200 Hz, 150 mA with 600 Hz, and 200 mA with 800 Hz were suitable for humane slaughter of chickens. Furthermore, Prinz et al. [21] observed that in a DC system, frequencies above 400 Hz did not produce suppressed or epileptiform EEG patterns, suggesting that very high frequencies may compromise stunning effectiveness. In the present study, oscilloscope measurements indicated that the water-bath stunner delivered unidirectional direct current (DC) with a relatively stable voltage output and minor voltage ripple. No periodic waveform characteristic of alternating current (AC) or pulsed DC was detected during the measurements. Because DC does not involve a defined oscillating frequency component, direct comparisons with previously published current–frequency recommendations for AC or pulsed DC systems are inherently limited. Nevertheless, the unconsciousness patterns observed at different current levels in the present study were generally consistent with the threshold current ranges reported in previous studies. These findings suggest that current intensity remains a critical determinant of stunning effectiveness, even when the electrical waveform differs from the AC or pulsed DC systems commonly evaluated in the literature.

Although unconsciousness generally increased at 140–160 V, the 120 V group showed lower unconsciousness rates than the 80 V group at P1. This deviation from a linear pattern may reflect biological variability and differences in body composition, as birds in the 120 V group had higher mean body weight and abdominal fat percentage and were slaughtered approximately 5 days later than the other groups, factors that may have influenced tissue impedance and consequently affected current delivery. Therefore, voltage effects should be interpreted in the context of delivered current rather than nominal voltage alone.

### 4.2. Body Composition, Breed and Stunning Effectiveness

Because the effectiveness of electrical water-bath stunning depends not only on electrical settings but also on bird-related biological characteristics, variation in body weight and body composition may play an important role in determining stunning outcomes. In the present study, body weight was positively correlated with abdominal fat weight, a relationship that is consistent with previous findings in commercial broiler strains. Latshaw and Bishop [34] reported a strong positive correlation between body weight and body fat content (R = 0.79) in Ross broilers, indicating that body weight is a reliable predictor of body fat and energy content. Similarly, Feddes et al. [35] demonstrated that carcass weight can serve as a robust indicator of body weight consistency in broilers.

In contrast, no association was detected between body composition and the duration of unconsciousness in the present study. This finding differs from the results of Rawles et al. [36], who reported that increasing live weight significantly shortened unconsciousness time in broilers stunned with a constant current. This discrepancy may be related to differences in breed, age, or body composition or stunning systems [8,9,10,37]. Earlier studies suggested that modern commercial broilers often exhibit higher whole-body fat content than some native or traditional strains [38], whereas the longer rearing periods and higher market weight of native chickens [9] might result in body composition differences, including a higher percentage of muscle or skeletal components than commercial broiler lines. However, more recent comparisons between commercial and slow-growing broilers raised under similar management have found higher carcass fat percentage in slow-growing strains than in commercial lines [39], indicating that fat deposition patterns are strongly strain- and system-dependent rather than universally lower in slow-growing or native chickens. Taken together, these findings imply that the relationship between body composition and stunning outcome is likely to depend on both breed and electrical setting. In our study, abdominal fat percentage was significantly associated with stunning effectiveness only at the lowest voltage (80 V; rho = 0.47, *p* < 0.01). At higher voltages (100–160 V), the observed correlations were weak (rho ranging from −0.16 to 0.03) and not statistically significant (*p* > 0.05). These findings indicate that body composition does not play a statistically meaningful role when sufficient electrical intensity is applied but may become influential when electrical conditions are close to the minimum threshold required to induce unconsciousness.

### 4.3. Behaviour Indicators as Practical Metrics of Unconsciousness

Although behavioural indicators are a relatively straightforward metric of consciousness, they must be combined with EEG analysis to evaluate whether each behaviour is indeed a reaction performed in an unconscious state, which ensures animal welfare at slaughter [32]. Our study examined the relationship between EEG-defined unconsciousness and behavioural indicators and evaluated how different electrical stunning conditions were associated with specific post-stunning behaviours. As voltage and current in-creased, the prevalence of spontaneous eye blinking and corneal reflexes decreased, whereas patterns of wing flapping and tonic–clonic activity showed more complex responses. Statistical analysis showed that tonic–clonic seizures and wing flapping were primarily affected by voltage, whereas neck tension was influenced by current (Table 4).

Similarly, Prinz et al. [30] reported that voltage affected eye reflexes and tonic–clonic activity, which were less frequent at high voltages, while wing flapping was not significantly influenced by voltage. Our results suggest that wing flapping also appeared to be less directly related to current intensity, although it may still be modulated by voltage. However, additional research is required to clarify the mechanisms by which electrical parameters influence this behaviour.

Previous studies have suggested that a corneal reflex prevalence below 30% can be considered an indicator of adequate stunning under commercial conditions [21,32]. Taken together, our findings suggest that higher electrical settings are more effective in suppressing spontaneous eye blinking and corneal reflexes and in inducing tonic–clonic activity, which is generally associated with a deeper level of unconsciousness.

### 4.4. Carcass Defects as Indirect Welfare Indicators

Carcass defects such as wing and breast haemorrhage are not only economic issues but may also indicate strong muscle contractions or cardiovascular disturbances associated with electrical stunning and can therefore be considered indirect indicators of compromised welfare. In the present study, the prevalence of wing and breast haemorrhage increased significantly with higher stunning voltages (*p* < 0.05). The highest level of wing and breast haemorrhage were observed in birds stunned at 160 and 140 V, respectively. According to Gregory and Wilkins [40], broilers stunned with currents at 130 mA showed increased breast haemorrhage. In our study, birds stunned at 160 V experienced a mean current of 130.5 ± 24.7 mA, which is consistent with this threshold.

Similarly, other researchers have found that applying currents exceeding 100 mA leads to a significant increase in breast haemorrhage [27] and bruised wing joints [41,42]. If stunning is ineffective, epileptic seizures and ventricular fibrillation may occur, which in turn can cause additional skeletal and muscular injuries and reduce meat quality [17]. In our experiment, the highest proportion of red wingtips and red pygostyle were recorded in the 160 V group, which may reflect impaired bleed-out. Several studies have indicated that high voltage in a water-bath stunner can induce ventricular fibrillation, resulting in inadequate bleeding, a high incidence of haemorrhaging, and even death before exsanguination [27,28,43,44,45]. Consistent with these findings, failure to achieve effective bleeding in birds with fibrillation has been associated with increased red wingtips [46] and red pygostyle [47].

Although higher voltage settings were generally associated with an increased prevalence of certain carcass defects [28], the relationship was not strictly linear across all voltage levels. Notably, the 120 V group exhibited higher rates of severe (Score 2) red wingtips and red pygostyle compared with the 140 V group. This pattern suggests that carcass damage may not depend solely on the magnitude of applied voltage but also on the consistency and depth of unconsciousness achieved.

Under constant-voltage conditions, insufficient current delivery, potentially resulting from higher apparent resistance, may lead to incomplete or inconsistent stunning [19,22]. Birds that are not fully unconscious immediately after stunning may exhibit stronger or more prolonged tonic and clonic muscle activity, wing flapping, or attempts to regain posture [17,48,49]. Such movements can increase mechanical trauma, vascular rupture, and localised haemorrhage in the wings and pygostyle region, thereby contributing to higher rates of severe red wingtips and tail bruising.

In contrast, more consistent induction of deep unconsciousness at 140–160 V may reduce vigorous post-stun movement, even though higher voltage itself can increase muscle contraction intensity during current application. Therefore, carcass defects likely reflect a complex interaction between electrical intensity and neuromuscular response, rather than a simple linear effect of voltage alone.

These findings emphasise that both insufficient and excessive electrical parameters can compromise outcomes, highlighting the importance of achieving consistent and adequately deep unconsciousness to balance animal welfare and carcass quality. Future studies could apply stratified randomization by body weight to further examine weight-dependent variation in resistance, current delivery, and stunning effectiveness under constant-voltage systems.

### 4.5. Limitations

The present results should not be interpreted as prescriptive stunning guidelines but rather as evidence highlighting the welfare trade-offs associated with different electrical settings in this breed. Several limitations should be considered when applying these findings to commercial practice. First, although oscilloscope measurements confirmed that the stunner delivered unidirectional direct current (DC), detailed electrical waveform characteristics beyond voltage output, such as ripple magnitude and stability during bird contact, were not continuously recorded during all stunning events. This limits precise comparison with regulatory recommendations that are typically expressed in terms of current and frequency for alternating current (AC) systems. Second, the experiments were conducted in a university slaughterhouse under controlled but not fully commercial conditions, and only 12–13-week-old female Taiwanese red-feathered native chickens were evaluated. Different ages, sexes, or breeds may require slightly different electrical settings to achieve optimal stunning efficacy and carcass quality. A slight difference in slaughter timing (approximately 5 days) occurred for the 120 V group due to logistical constraints. Although this falls within normal commercial variation, it may have contributed to variability in body composition and apparent resistance. Third, because EEG recording began at 10 s post-stun, the immediate onset of unconsciousness during the first seconds after stunning was not directly assessed. The present findings, therefore, primarily reflect the persistence and duration of unconsciousness rather than its exact induction time. Finally, behavioural observations were performed by a single trained observer, which ensured consistency but did not allow assessment of inter-observer reliability. Despite these limitations, the present findings provide useful evidence to inform welfare assessment of stunning effectiveness in Taiwanese red-feathered native chickens under commercial slaughter conditions, while also highlighting the trade-off between prolonged unconsciousness and increased carcass damage at higher electrical settings.

### 4.6. Implications

This study demonstrates that electrical water-bath stunning conditions developed for commercial broilers may not reliably ensure unconsciousness in native or slow-growing poultry breeds. Linking EEG-defined unconsciousness with readily observable behavioural indicators provides a welfare-relevant framework for evaluating stunning effectiveness under field conditions. The strong association between EEG-based unconsciousness and the suppression of corneal reflex and spontaneous eye blinking highlights the value of these indicators for on-site welfare monitoring when direct neurophysiological measurements are not feasible.

At the same time, the increased prevalence of carcass defects at higher electrical settings underscores the existence of a welfare trade-off related to excessive neuromuscular responses. These findings emphasise the importance of assessing stunning effectiveness using multiple welfare-relevant outcomes rather than relying solely on electrical settings, and they support the need for breed-specific evaluation of stunning practices in poultry slaughter systems.

From a veterinary perspective, these findings provide objective evidence to support decision-making during slaughterhouse supervision, particularly for evaluating unconsciousness and stunning effectiveness in slow-growing poultry.

## 5. Conclusions

Taiwanese red-feathered native chickens respond differently to electrical water-bath stunning than commercial broilers, and the direct application of broiler-based settings may compromise animal welfare. EEG recordings confirmed that higher voltages (140–160 V) increased the likelihood of unconsciousness, while corneal reflex and spontaneous eye blinking reliably reflected EEG-defined unconsciousness and can serve as practical on-site welfare indicators. However, higher voltages were also associated with an increased prevalence of carcass defects, indicating a welfare trade-off linked to excessive neuromuscular responses. These findings emphasise the need to evaluate stunning effectiveness in slow-growing poultry breeds using both physiological and behavioural indicators, rather than relying solely on electrical settings developed for commercial broilers.

## Figures and Tables

**Table 1:** Means and SD of electrical conditions and body composition.

Voltage (V)	N	Electrical Conditions		Unconscious Time (s)	Body Composition			
		Current (mA)	Stunning Time (s)		BW ^1^ (±SD) (kg)	AFW ^1^ (±SD) (kg)	AFW% ^2^	CW ^1^ (±SD) (kg)
80	38	42.6 ± 15.5 ^d^	7 ± 0.4 ^a^	35.1 ± 23.9 ^bc^	2.62 ± 0.27 ^c^	0.09 ± 0.04 ^bc^	3.49 ± 1.42 ^bc^	2.13 ± 0.22 ^c^
100	38	88.7 ± 16.6 ^c^	6.4 ± 0.6 ^b^	26.5 ± 18.3 ^c^	2.62 ± 0.23 ^c^	0.08 ± 0.03 ^c^	3.2 ± 1.22 ^c^	2.13 ± 0.16 ^c^
120	33	76.3 ± 45.9 ^c^	6.3 ± 0.5 ^b^	24.4 ± 14.6 ^c^	3.08 ± 0.32 ^a^	0.13 ± 0.04 ^a^	4.07 ± 1.22 ^ab^	2.58 ± 0.25 ^a^
140	38	117.1 ± 25.3 ^b^	7 ± 0.4 ^a^	44.1 ± 25.0 ^ab^	2.85 ± 0.37 ^b^	0.13 ± 0.04 ^a^	4.46 ± 1.28 ^a^	2.31 ± 0.26 ^b^
160	39	130.5 ± 24.7 ^a^	7 ± 0.5 ^a^	49.6 ± 21.7 ^a^	2.69 ± 0.42 ^b^	0.11 ± 0.06 ^b^	3.78 ± 1.64 ^bc^	2.16 ± 0.3 ^c^
*p*-value		<0.001	<0.001	0.0001	<0.001	0.0002	0.0010	<0.001

^1^ BW: body weight (kg), AFW: abdominal fat weight (kg) and CW: carcass weight (kg). ^2^ AFW% = $\frac{Abdominal\;fat\;weight\;(kg)}{Body\;weight\;(kg)}\times 100$. ^a,b,c,d^ Means significantly different among voltage groups (one-way ANOVA followed by Tukey’s post hoc test, *p* < 0.05).

**Table 2:** Pearson’s correlation between body composition and duration of unconsciousness.

Variables	BW (kg)	AFW (kg)	CW (kg)	Unconscious Time (s)
BW (kg)	-			
AFW (kg)	0.656 **	-		
CW (kg)	0.947 **	0.575 **	-	
Unconscious time (s)	0.056	0.082	−0.116	-

BW; body weight (kg), AFW; abdominal fat weight (kg), and CW; carcass weight (kg). ** Correlation is significant at the 0.01 level (2-tailed).

**Table 3:** Percentage of EEG states at different voltages.

Time Point	EEG Pattern	80 V (*n* = 32)	100 V (*n* = 29)	120 V (*n* = 20)	140 V (*n* = 36)	160 V (*n* = 36)
P1	Profound reduction	11(34%)	12 (41%)	8 (40%)	8 (40%)	8 (40%)
	Epileptiform	5 (16%)	5 (16%)	5 (16%)	5 (16%)	5 (16%)
	Total unconscious	16 (50%)	16 (50%)	16 (50%)	16 (50%)	16 (50%)
P2	Profound reduction	10 (31%)	10 (31%)	10 (31%)	10 (31%)	10 (31%)
	Epileptiform	5 (16%)	5 (16%)	5 (16%)	5 (16%)	5 (16%)
	Total unconscious	15 (47%)	15 (47%)	15 (47%)	15 (47%)	15 (47%)
P3	Profound reduction	5 (16%)	5 (16%)	5 (16%)	5 (16%)	5 (16%)
	Epileptiform	4 (13%)	4 (13%)	4 (13%)	4 (13%)	4 (13%)
	Total unconscious	9 (28%)	2 (7%)	6 (30%)	10 (30%)	15 (42%)

Periods: P1, 10 to 20 s; P2, 20 to 30 s; P3, 30 to 40 s. Effects of electroencephalogram (EEG) and electrical conditions were analysed using a Chi-squared distribution. Profound reduction: less than 10% of pre-stunning brain power. Epileptiform: waveform shows high amplitude and frequency.

**Table 4:** Association between EEG and electrical conditions.

Factor Effect	No. of Unconscious Chicken					*p*-Value	
	80 V	100 V	120 V	140 V	160 V	Voltage	Current
P1	16	12	8	27	29	<0.001	0.003
P2	15	8	5	14	28	<0.001	0.316
P3	9	2	6	10	15	0.043	0.073

Periods: P1, 10 to 20 s; P2, 20 to 30 s; P3, 30 to 40 s.

**Table 5:** Association between behavioural responses and electrical conditions.

Factor Effect	*p*-Value	
	Voltage	Current
Spontaneous eye blinking P1	0.003	<0.001
Spontaneous eye blinking P2	<0.001	0.006
Spontaneous eye blinking P3	0.008	0.322
Corneal reflex P1	<0.001	<0.001
Corneal reflex P2	<0.001	0.002
Corneal reflex P3	0.001	0.029
Wing flapping	<0.001	0.831
Neck tension	0.063	0.034
Tonic–clonic	<0.001	0.016

Periods: P1, 10 to 20 s; P2, 20 to 30 s; P3, 30 to 40 s.

**Table 6:** Linear correlation between stunning effectiveness and AFW%; abdominal fat % in different voltage group.

	Spearman’s Rho	AFW%
EEG 80 V (N = 9)	Correlation coefficient	0.47
	*p*-value	<0.01
EEG 100 V (N = 2)	Correlation coefficient	−0.16
	*p*-value	0.4
EEG 120 V (N = 4)	Correlation coefficient	−0.13
	*p*-value	0.6
EEG 140 V (N = 10)	Correlation coefficient	0.03
	*p*-value	0.8
EEG 160 V (N = 13)	Correlation coefficient	−0.1
	*p*-value	0.6

EEG 80 V: overall EEG from birds stunned with 80 V; EEG 100 V: overall EEG from birds stunned with 100 V; EEG 120 V: overall EEG from birds stunned with 120 V; EEG 140 V: overall EEG from birds stunned with 140 V; and EEG 160 V: overall EEG from birds stunned with 160 V; AFW% = $\frac{Abdominal\;fat\;weight\;(kg)}{Body\;weight\;(kg)}\times 100.$

**Table 7:** Effect of electrical condition on carcass defect expressed as percentages.

Voltage (V)	N	WHM			BRHM			RWT			RPG		
		0	1	2	0	1	2	0	1	2	0	1	2
80	38	50.0	47.4	2.6	44.7	44.7	10.5	28.9	71.7	0.0	47.4	44.7	7.9
100	38	65.8	31.6	2.6	39.5	50.0	10.5	55.3	44.7	0.0	31.6	65.8	2.6
120	33	45.5	51.5	3.0	42.4	48.5	9.1	33.3	57.6	9.1	48.5	39.4	12.1
140	38	39.5	50.0	10.5	15.8	73.7	10.5	36.8	55.3	7.9	42.1	57.9	0.0
160	39	28.2	71.8	0.0	20.5	61.5	17.9	25.6	66.7	7.7	25.6	64.1	10.3
*p*-value		0.014			NS			0.025			0.043		

WHM: Wing haemorrhage, BRHM: Breast haemorrhage, RWT: Red wingtips, and RPG: Red pygostyle; 0: Indicates absence of damage; 1: Indicates slight damage presence; 2: Indicates severe damage presence. NS: Not significant.

## Data Availability

The original contributions presented in this study are included in the article and Supplementary Materials. Further inquiries can be directed to the corresponding author.

## Supplementary Materials

The following supporting information can be downloaded at: https://www.mdpi.com/article/10.3390/vetsci13030273/s1, Figure S1: Oscilloscope verification of the electrical output waveform of the commercial water-bath stunner used in the experiment. Measurements were obtained using a digital oscilloscope (DSOX1202A, Keysight Technologies, Santa Rosa, CA, USA). The recorded signals showed unidirectional direct current (DC) output with minor voltage ripple and no detectable pulsed waveform. (A) Nominal setting 80 V. Measured RMS voltage ≈ 82.9 V with peak-to-peak ripple ≈ 10.9 V. (B) Nominal setting 100 V. Measured RMS voltage ≈ 100.9 V with peak-to-peak ripple ≈ 18.1 V. (C) Nominal setting 120 V. Measured RMS voltage ≈ 120.5 V with peak-to-peak ripple ≈ 24.1 V. (D) Nominal setting 140 V. Measured RMS voltage ≈ 140.0 V with peak-to-peak ripple ≈ 14.1 V. The waveform at the nominal 160 V setting was not recorded during oscilloscope measurements.

## Abbreviations

The following abbreviations are used in this manuscript:

EEG	Electroencephalography
FFT	Fast Fourier Transform
BW	Body weight
AFW	Abdominal fat weight
CW	Carcass weight
BHM	Breast haemorrhage
RPG	Red pygostyle
WHM	Wing haemorrhage
RWT	Red wingtips
