# Supplementary material for: Water-Bath Stunning Efficiency, Welfare Indicators, and Carcass Quality in Taiwanese Red-Feathered Native Chickens
Source: Vet Sci. 2026 Mar 16;13(3):273. doi: 10.3390/vetsci13030273 (PMC13029926; doi:10.3390/vetsci13030273)
Supplement: Supplementary file 1 [file vetsci-13-00273-s001.zip › vetsci-4150367-supplementary.pdf]

(A)

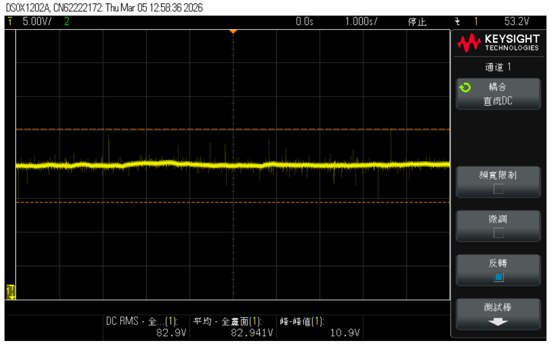

(B)

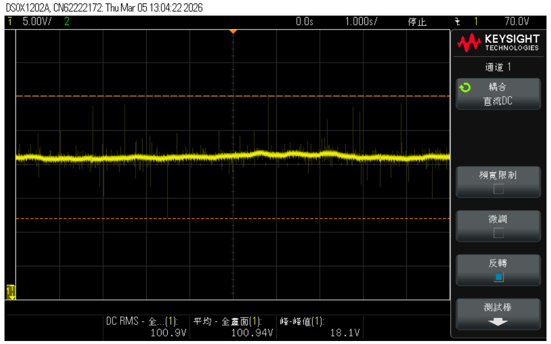

(C)

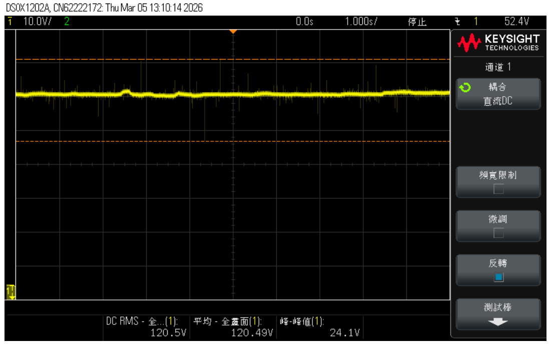

(D)

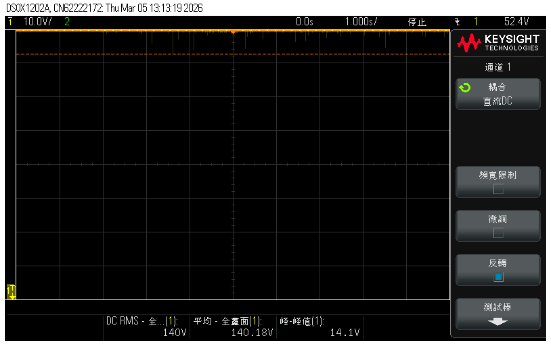

Supplementary Figure S1. Oscilloscope verification of the electrical output waveform of the commercial water-bath stunner used in the experiment. Measurements were obtained using a digital oscilloscope (DSOX1202A, Keysight Technologies, Santa Rosa, CA, USA). The recorded signals showed unidirectional direct current (DC) output with minor voltage ripple and no detectable pulsed waveform.(A) Nominal setting 80 V. Measured RMS voltage ≈ 82.9 V with peak-to-peak ripple ≈ 10.9 V. (B) Nominal setting 100 V. Measured RMS voltage ≈ 100.9 V with peak-to-peak ripple ≈ 18.1 V. (C) Nominal setting 120 V. Measured RMS voltage ≈ 120.5 V with peak-to-peak ripple ≈ 24.1 V. (D) Nominal setting 140 V. Measured RMS voltage ≈ 140.0 V with peak-to-peak ripple ≈ 14.1 V. The waveform at the nominal 160 V setting was not recorded during oscilloscope measurements.
